# Transparency and adaptability aid in realigning the complexity of objectives, approaches, and systems in human-wildlife coexistence research

**DOI:** 10.1038/s41598-024-69563-5

**Published:** 2024-09-17

**Authors:** Claire F. Hoffmann, Jacalyn M. Beck, Roselyn W. Kaihula, Robert A. Montgomery

**Affiliations:** 1 Yale Center for Biodiversity and Global Change, Yale University, 165 Prospect Street, New Haven, CT 06520 USA; 2 American Conservation Experience, 2900 N. Fort Valley Rd, Flagstaff, AZ 86001 USA; 3 World Bank, 50 Mirambo Street, P. O. Box 2054, Dar es Salaam, Tanzania; 4 Department of Biology, University of Oxford, 11a Mansfield Road, Oxford, OX1 3SZ UK

**Keywords:** Complex systems, Human-wildlife coexistence, Transparency, Research approaches, Conservation biology, Environmental social sciences

## Abstract

Human-wildlife interactions are situated within dynamic systems, characterized by social and ecological complexity. Human-wildlife coexistence research, however, typically focuses on one component of these systems in isolation. We inadvertently followed this norm while carrying out semi-structured interviews of livestock-owners in Northern Tanzania. As existing literature highlighted that this area was a hotspot for livestock depredation, our research questions focused on human interactions with carnivores. Interestingly, almost three quarters (72%, *n* = 72 of 100) of study participants independently raised African elephants (*Loxodonta africana*) as presenting the greatest impediments to coexistence. By centering our interviews on carnivores, we omitted vital components of this complex system. To counteract the effects of this oversimplification, we changed our intended analytical process after data collection. Instead of conducting a quantitative analysis of rates of livestock depredation and perceptions of risk posed by a suite of sympatric carnivores, we applied a grounded theory approach to assess interactions across multiple dimensions of this complex system. Through this transparent effort to realign our approaches with the complexity of the study system, we highlight the importance of designing research approaches that effectively reflect the complexities inherent to human-wildlife coexistence.

## Introduction

Complex systems approaches are increasingly applied to conceptualize a variety of conservation challenges^1,2^. The four key features commonly used to define complex systems include: (*i*) the presence of numerous interacting components, (*ii*) constant change, (*iii*) the lack of clear boundaries to the system, and (*iv*) feedback loops that can yield unpredictable patterns^1,3^. Systems featuring integrated social and ecological components are often termed coupled human and natural systems (CHANS)^2^. Most contemporary conservation challenges exhibit the characteristics of complex CHANS, with interacting social components such as development needs or colonial histories, and ecological components such as trophic cascades or changing climatic regimes^2–4^. Thus, explorations of human-wildlife coexistence are particularly well-suited to complex systems approaches^3,5–7^.

Human-wildlife coexistence refers to a dynamic state of harmony between humans and wildlife^8^. While often distilled down to addressing competition for resources, in practice coexistence is much more intricate and complex^4,9,10^. For example, religious beliefs^11,12^, patterns of land use change^13^, social and economic inequalities^9^, emotions^14–16^, and inter-species interactions^17^ have all been shown to impede human-wildlife coexistence. Therefore, research designed to promote human-wildlife coexistence must incorporate a range of social and ecological components^6,18^. Detailed theoretical and methodological outlines have been proposed to apply a complex systems approach to human-wildlife coexistence research^4,18,19^. However, studies in this field still tend to focus on only one component of the complex system at a time^19,20^. This trend is not dissimilar from tendencies to simplify complex systems across many types of ecological research^20^. Furthermore, human-wildlife coexistence research commonly explores one component via single-species or single-guild case studies^19,21,22^. As a result, prevailing knowledge of human-wildlife coexistence systems is often fragmented, with deep domain expertise of comparatively narrow species-specific components^18,19^.

We followed this tendency by designing a study that inadvertently simplified human-wildlife coexistence. We conducted 100 semi-structured interviews with small-scale agropastoralists affected by high rates of livestock depredation in Northern Tanzania. Given that the existing literature had identified this system as a depredation hotspot^23,24^, we designed an interview protocol that combined two separate sets of questions, with the core of the study focusing on the consequences of conflict from carnivores alone. The other set of questions assessed community involvement in the management of wildlife. Although we presented the two question sets in the same interview session, we intended for them to be entirely separate studies combined only for logistical and ethical reasons. During open-ended portions of these interviews, participants repeatedly redirected our questions to discuss African elephants (*Loxodonta africana*; hereafter ‘elephants’), which they believed had a much greater influence on human-wildlife coexistence. Upon completing these surveys, we realized that we had too narrowly focused on human-carnivore interactions and omitted human-elephant interactions, as well as conflicts with researchers and government officials associated with elephant interactions, as key components of the complex system.

There is a critical need for increased openness regarding reflexive practices and critical lessons learned during conservation research, particularly in the context of complex systems^25–27^. As multiple components of these systems may be facing urgent threats, it is essential that resources for researching conservation challenges are used efficiently^28^. Sharing missteps in research design helps prevent repetition of the same unsuccessful outputs, improves institutional knowledge, advances innovation in research methods, and can even stimulate more learning transfer and emotional engagement than communication of successes^25,26^.

To promote greater transparency in human-wildlife coexistence research, here we communicate openly about how we adapted our strategy to better incorporate complex systems approaches. We dropped our initial intention to carry out a quantitative analysis of the carnivore-specific interview questions in isolation, assessing participant perceptions of the risk posed by each carnivore species present in their communities. Instead, we conducted a comprehensive analysis of the complete interview data via constant comparative analysis to detect emergent themes regarding the intersecting impacts of human-elephant and human-carnivore interactions. We explored how these themes embodied the intersections between components of the complex systems in which human-wildlife coexistence is situated. We discuss how this approach was able to reveal deep understanding of the impediments to human-wildlife coexistence in our study site, and of the complex system itself. We highlight the importance of research design that can drive more nuanced understanding of human-wildlife coexistence in complex systems.

## Methods

### Study site

**Figure 1:**
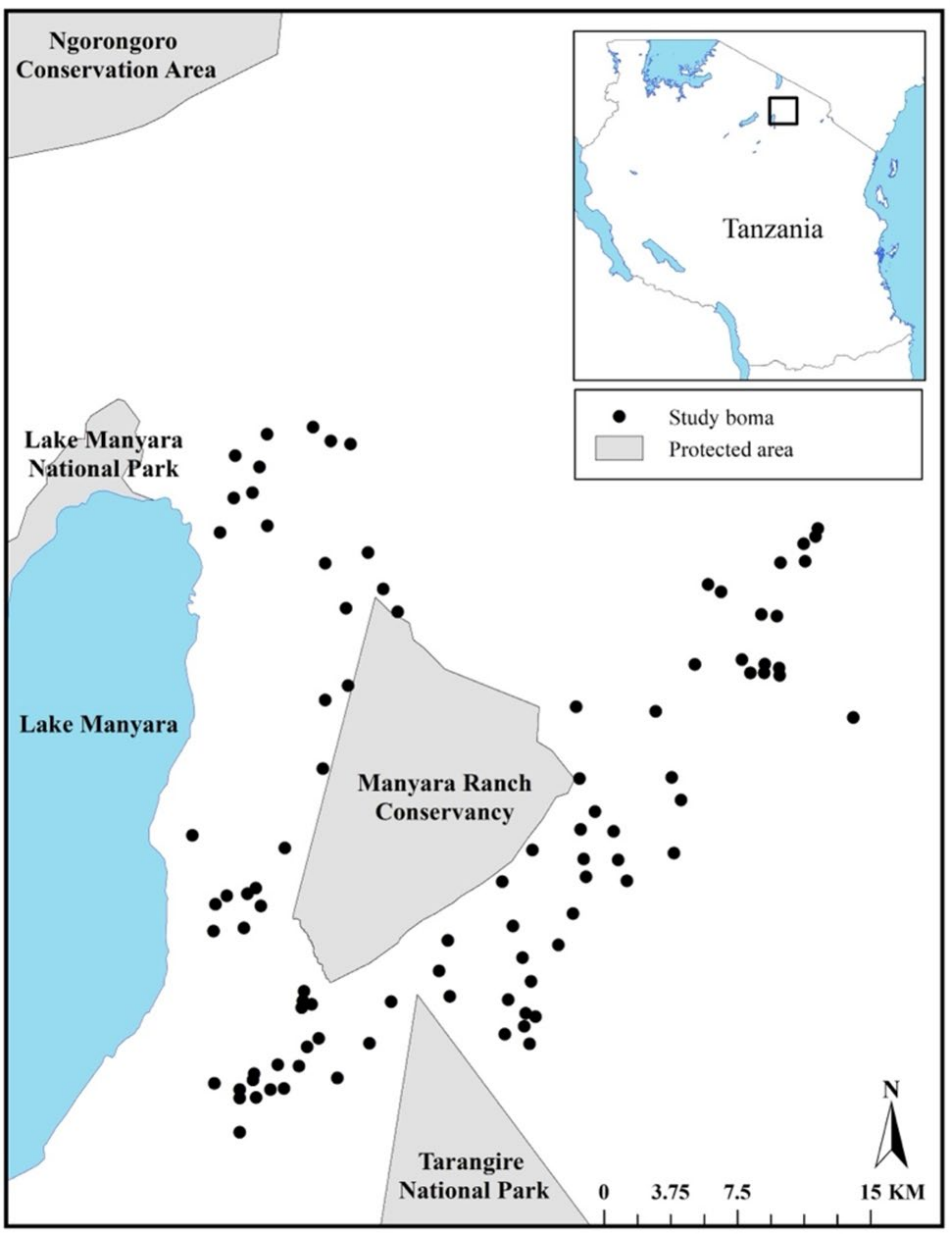
The location of the 100 bomas included in semi-structured interviews about human-wildlife coexistence in Northern Tanzania from May to August 2018.

We positioned our study in Northern Tanzania, in a system comprised of a matrix of protected areas and human community lands^29^. The study area contains four primary protected areas including Lake Manyara National Park, Tarangire National Park, Manyara Ranch Conservancy, and Ngorongoro Conservation Area (Fig. 1). The system supports a population of approximately 640,000 people, growing at an average rate of 3.4% per year^30,31^. The local people in this system primarily maintain agropastoralist lifestyles with small scale agricultural crops and livestock including cows, sheep, and goats^24,29^. Maasai, Waarusha, Ndorobo are the most common ethnic groups in the region, with smaller populations of Barbaig, Datoga, Pare, Hadzabe, Sandawe, Sonjo, Chagga, Fipa, Nyaturu, and Iraqw^32^. The system also supports populations of carnivores such as African lions (*Panthera leo*; hereafter ‘lions’), African leopards (*Panthera pardus*; hereafter ‘leopards’), spotted hyenas (*Crocuta crocuta*), African wild dogs (*Lycaon pictus)*, and black-backed jackals (*Canis mesomelas*)^24,30^ as well as diverse guilds of browsing and grazing herbivores including elephants, plains zebras (*Equus quagga*), and common wildebeest (*Connochaetes taurinu*s)^33,34^. This region experiences some of the highest rates of livestock depredation in the world^23^, due to high spatial overlap between livestock owners and carnivores, high density of carnivores across much of the landscape, and low rates of active herding strategies^24,35^.

### Semi-structured interviews

We centered our examination on ten focal villages across our study system (Mbuyuni, Esilalei, Losirwa, Makuyuni, Minjingu, Mswakini Chini, Mswakini Juu, Naiti, Naitolia, Olasiti, and Oltukai; Fig. 1). We selected households (i.e., bomas) for inclusion in our study via a stratified random sample designed to ensure geographic distribution across the extent of our study area. Applying these same principles, we then selected ten bomas per village, with at least one boma in each of the spatially distinct areas (i.e., sub-villages). Our field team consisted of CFH, RWK, and a Tanzanian field assistant. Both RWK and the field assistant are fluent in English and Swahili, commonly spoken among these rural communities. In each village, we also hired a local guide to help us locate bomas, introduce us to the head of each household, and provide additional translation assistance if the participant preferred to conduct the interview in Maa, the most common local language. Upon arriving at a potential boma, the local guide approached the head of the household to introduce the research project. We then received verbal informed consent from the respondent to conduct the interviews and captured all answers with notations and audio recordings. In instances in which the male head of the household was not present, we asked if the head of the household would be willing to participate. One female head of household declined to participate in the interview, but all other potential participants agreed. The majority of our interview participants (80%, *n* = 80) were male, with the remaining 20 (20.0%) female. We implemented the interviews in Maa and translated the participants’ responses to Swahili in real time to ensure the notations and recordings were interpretable at a later date.

We carried out all procedures in accordance with the relevant guidelines and regulations. Prior to conducting the study, all procedures were approved by Michigan State University’s Institutional review board on the use of human subjects (IRB # i053842) and were fully permitted and approved both by the Tanzanian national parks authority (TANAPA) and the Tanzanian wildlife research institute (TAWIRI). We also received written permission from the Tanzanian government at the regional, district, ward, and village levels prior to carrying out the interviews. We followed a semi-structured protocol, with our interviews separated into three sections.

We initiated the interviews with open-ended questions related to daily life and community, including queries regarding: (*i*) background information (e.g., ‘can you describe what your daily life is like?’) and (*ii*) resources (e.g., ‘What resources are available in this area?’)*.* We differentiated communities from villages, in that we considered communities to be social groups with no institutionalized boundaries. Villages, in contrast, referred to the administrative locality derived from the hierarchical Tanzanian government structure. In doing so, we allowed participants to determine which individuals were relevant for inclusion in their responses, regardless of which village they lived in. We then transitioned to human-carnivore interactions, specifically inquiring about participants’ perceptions of how often carnivores were present at their bomas and how often the carnivores attacked their livestock using a five-point Likert scale (never, rarely, sometimes, often, very often). We also asked which carnivore they perceived to pose the greatest risk to their livestock and livelihoods, and how many of each livestock species had been depredated by carnivores in the past year. In the final interview section, we asked about perceptions of wildlife management, including questions on (i) wildlife conservation (e.g., ‘What do you think are the benefits of wildlife conservation for the community?’), (ii) wildlife management (e.g., ‘Do community members participate in decisions regarding wildlife?’), and (iii) an opportunity for participants to introduce any additional topics that we did not cover in the interview questions (see Appendix 1 for a full list of interview questions).

Our interview sections were intended to serve as two separate areas of inquiry, with independent analytical approaches and applications. We combined them into one single interview session to streamline field logistics and avoid research fatigue in our participating communities. Half of the interview was informed by social work theories, with the goal of exploring the intersection between social challenges and environmental resources. We developed the second section of the interview, in contrast, to focus in on carnivores. Specifically, we intended to assess human-carnivore coexistence, with an emphasis on livestock depredation. Overall, we designed the semi-structured interview with a combination of closed and open-ended questions so that participants could introduce new topics of conversation. We did not, however, expand on those avenues of inquiry beyond the information that was volunteered. After completion of all semi-structured interviews, we translated the audio recordings into English and fully transcribed each recording.

In the process of implementing these 100 semi-structured interviews, it became evident that impediments to human-carnivore coexistence within this system extended beyond carnivore depredation of livestock alone. Interview participants clearly indicated that human-wildlife coexistence more broadly was being negatively impacted by components of the system that we had not included in our interview questions or had not intended to capture in our analysis. Specifically, participants repeatedly discussed their interactions with both carnivores and elephants in the same response, even to questions that did not reference either type of animal. In fact, none of our interview questions introduced the topic of human-elephant interactions, yet our participants discussed the intersection of elephant and carnivore impacts throughout all sections of the interview. Therefore, impediments to coexistence with carnivores and elephants were intrinsically linked in this system. Based upon this realization, we centered our complex systems approach on the intersection between elephant and carnivore-related components. We also determined that we could not explore these intersections within the system without analyzing all three interview sections together.

**Figure 2:**
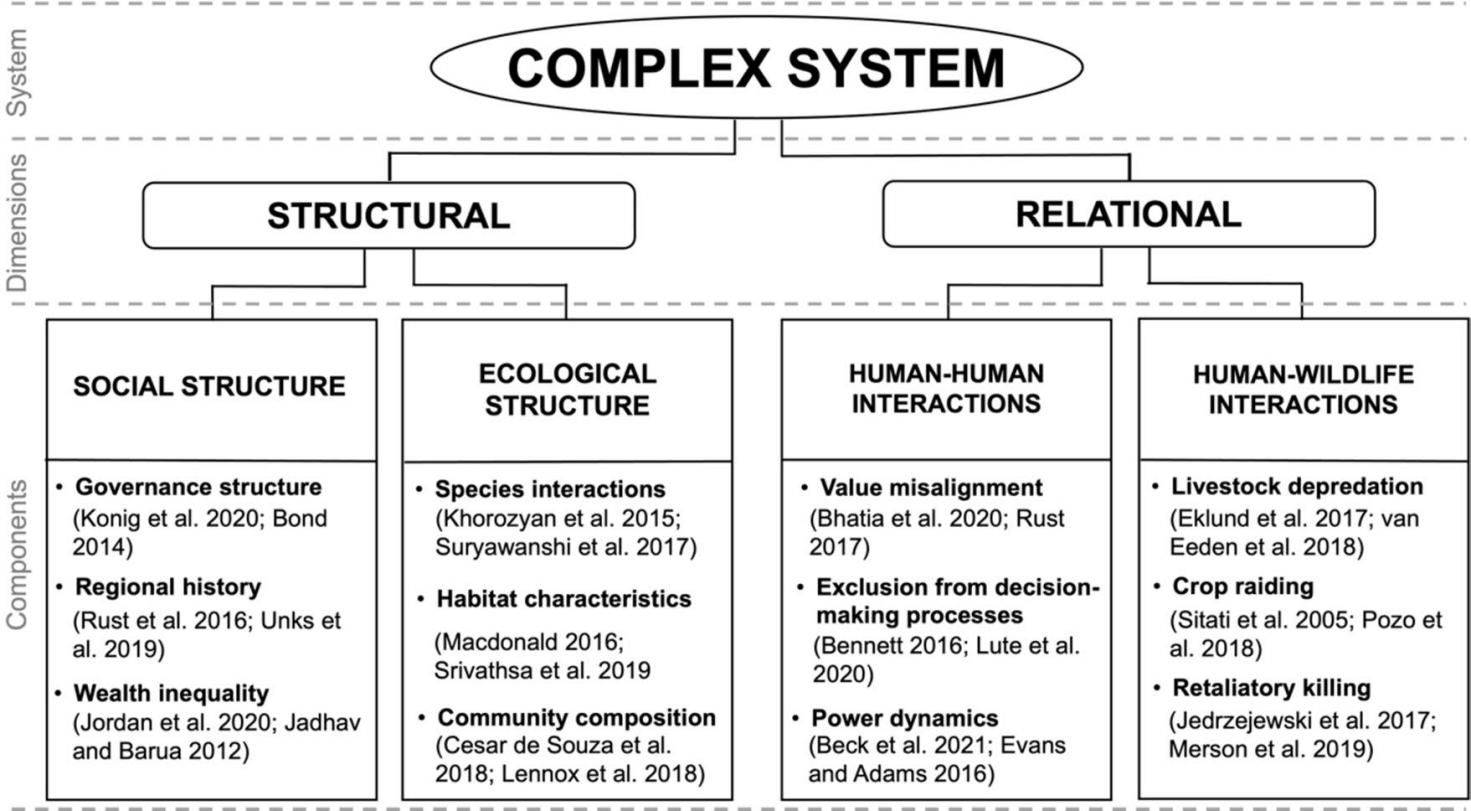
A conceptual diagram illustrating some of the potentially interacting components of the complex systems in which human-wildlife coexistence is situated. The systems can be divided into a structural dimension, containing social and ecological components, and a relational dimension containing human-human and human-wildlife components. A non-exhaustive list with representative examples of each type of component are provided, along with supporting citations.

**Figure 3:**
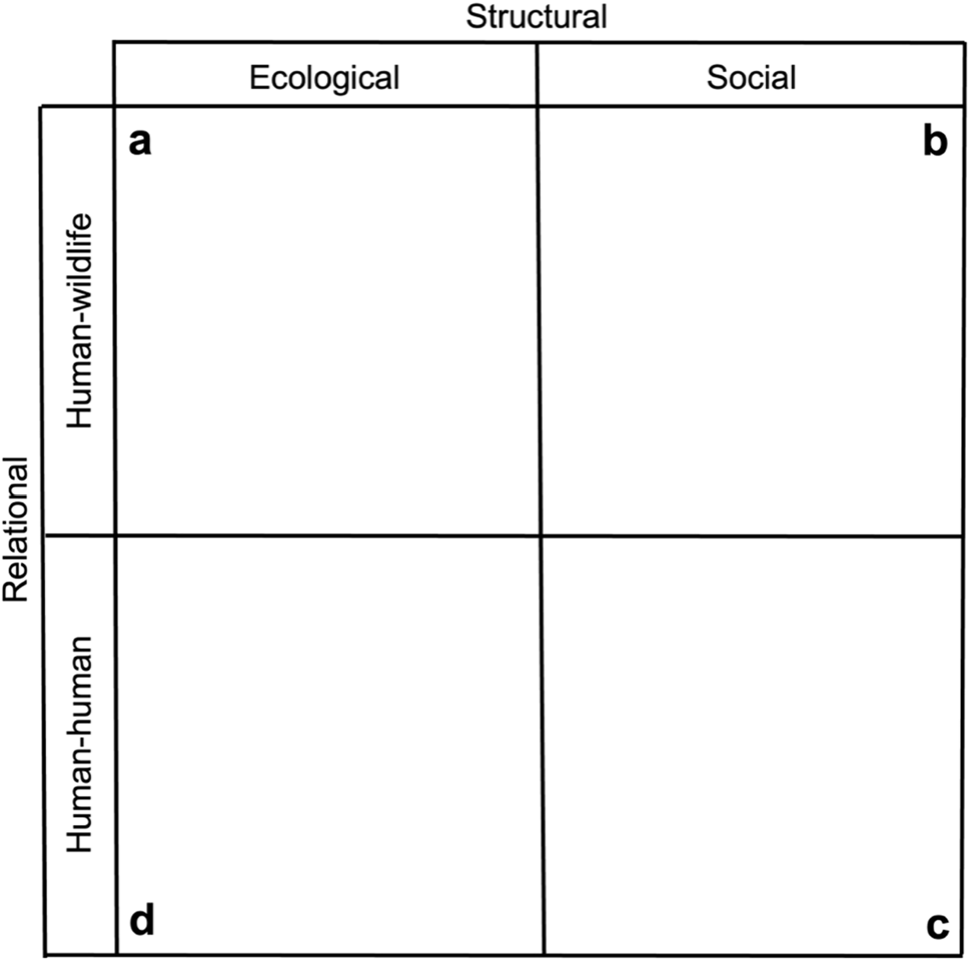
A framework of the complex system dimensions and components, for use in visualizing intersections within the system.

As a complex CHANS, our study system includes a multitude of interacting components. To aid in conceptualizing this system, we divided the components among two dimensions based upon common categorizations of system components in the published literature (Fig. 2). The first dimension contains the system’s structural components, which we categorized as either social or ecological^3,18^. The social components include political histories, economic systems, and administrative structures, among others^9,10^. The ecological components include climatic regimes, species diversity, migratory patterns, and any other factors that may contribute to the ecological structure of the system^36^. The second dimension is relational, and contains components of the interactions between various actors in the system^37,38^. We followed the established definition of actors as individuals (both human and non-human), institutions, or entities that have the capacity to influence systems-level values and actions ^39,40^. We categorized the relational components as either human-human or human-wildlife. Human-human interactions include relational components like conflicts arising from contradictory interests related to wildlife as well as collaborations between communities to achieve a shared goal^6,19,41^. We categorized interactions such as carnivore depredation of livestock and coupled retaliatory killing of carnivores as human-wildlife components^22,37^. These relational components are often framed as conflicts. However, this negative framing is increasingly recognized as overly simplistic and detrimental to coexistence^38,41–43^. Therefore, we refer to these components as relational to avoid limiting our assessment to purely negative interactions among actors. Finally, to aid in visualizing the interactions between components of our system, we created a framework of these intersecting dimensions and components (Fig. 3).

We then used grounded theory to analyze the transcripts from the semi-structured interviews^44,45^. Grounded theory is an analytic approach that avoids applying preconceived ideas about the topic, instead allowing themes and theories to be derived from the content under study during data collection and analysis^45,46^. Within this grounded theory approach, we analyzed the interview transcripts using the constant comparative analysis method^45,47^. Via this process, we conducted two rounds of coding, the first to classify initial codes and the second to quantify patterns and themes among those codes^47^. We conducted this analysis in triplicate, with CFH, JMB, and RWK each independently coding the transcripts in Dedoose 8.7 (Socio-Cultural Research Consultants, LLC, Los Angeles, CA). We then discussed our codes and rationales until we came to a collective agreement on the final emergent themes. Each team member then independently re-analyzed the interviews to quantify the frequency of the six emergent themes.

Next, we assessed theme co-occurrence via a multi-step process. Here, we define co-occurrence to be an instance in which we identified more than one theme as being present within a single interview. First, by working on an interview-by-interview basis, we determined which themes were present and created a pairwise list of co-occurring themes. For example, if one interview contained the themes A, B, and C, we considered A/B to co-occur, as well as both A/C and B/C. Second, we calculated the total number of co-occurrences for each pair of themes, across all interviews. In other words, we quantified the number of times A/B co-occurred total, as well as A/C, and so on for all co-occurrence pairs. Finally, we assessed whether this co-occurrence number was > 50% of the total number of occurrences for either theme within a given pair. While all co-occurrences were inherently reciprocal, each theme had different numbers of total occurrences, so the 50% cutoff may have rendered some co-occurrence patterns unidirectional. As a concrete example, assume there were 20 occurrences of theme A, 30 occurrences of theme B, and 11 co-occurrences of A/B. Theme A would have > 50% co-occurrence with theme B, but the reverse would not be true. We selected 50% as our cutoff to indicate whether the co-occurrences represented the majority of occurrences for any given theme. Overall, the > 50% co-occur4ence value represents intersections of themes within each interview and is indicative of the relationships between system components in the personal experiences of our participants.

We also explicitly assessed how the six themes represented intersecting components from multiple dimensions of the complex system (i.e., social structure, ecological structure, human-human interactions, human-wildlife interactions). We determined these intersections based on the content of the interview excerpts that we coded as falling within each theme. As an example, many excerpts within the theme ‘threat to food security’ centered around the prevalence of elephant crop raiding. Crop raiding is a human-wildlife interaction strongly influenced by the ecological structures of the system^48^ and thus represents the intersection of those two types of components. We mapped the intersecting components of each theme and the patterns of theme co-occurrence through a visual representation of the two dimensions.

In 72 of the interviews conducted, the participants brought up coexistence with elephants although no interview questions specifically addressed that species. To explore this interaction and its implications for the intersection of components in the complex system, we narrowed in on this sample of 72 interviews for our complex systems approach. This decision is consistent with a grounded theory approach, as we allowed our analysis to be informed by an observed pattern to best represent the phenomenon under study, as opposed to maintaining an approach based on preconceived notions^44,45^.

## Results

We conducted 100 interviews at bomas distributed across 43 sub-villages. We excluded one interview from our analysis, as we discovered in the midst of conducting the interview that the participant did not own livestock. Therefore, we concluded the interview process but determined his answers would not be comparable to those of our other participants. Finally, following the grounded theory approach, we analyzed 72 of the remaining 99 interviews. Among those 72 interviews, 52 participants (72.2%) were male, and the other 20 (27.8%) were female.

We identified 74 initial codes in the first iteration of analysis, and six emergent themes in the second (Tables 1, 2). The themes were: (*i*) threats to food security, (*ii*) threats to human safety, (*iii*) threats to societal well-being, (*iv*) need for wildlife education, (*v*) need for trust in government, and (*vi*) need for resources and solutions (Table 2). We identified a total of 409 occurrences of these six themes. The most common theme was ‘need for resources and solutions’ (*n* = 116, 28.4%), followed by ‘threats to food security’ (*n* = 94, 23.0%), ‘need for trust in government’ (*n* = 82, 20.0%), ‘need for wildlife education’ (*n* = 49, 12.0%), ‘threats to societal well-being’ (*n* = 49, 12.0%), and ‘threats to human safety’ (*n* = 19, 4.6%; Table 3).

**Table 1:** The codes and themes identified via constant comparative analysis of 72 interviews on human-wildlife coexistence in Northern Tanzania. The codes and themes are specific to elephants (*Loxodonta africana*), as informed by our active listening process during data collection. The codes shown here are representative examples of those subsequently consolidated into emergent themes.

First iteration: initial codes		
1A. Crop loss	1B. Threat to human safety	1C. Hunger
1A. Farm destruction	1B. Elephants kill people	1C. Poverty
1A. Wildlife attack (farms)	1B. Wildlife attack (people)	1C. Societal impact
2A. Education	2B. Elephants more important than people	2C. Farm protection strategies
2A. Lack of solutions	2B. Compensation	2C. No solutions/help
2A. Community meetings about problems	2B. No follow through on promised help	2C. Human-elephant conflict mitigation

Second iteration: emergent themes		
1A. Threat to food security	1B. Threat to human safety	1C. Threat to societal well-being
2A. Need for wildlife education	2B. Need for trust in government	2C. Need for resources and solutions

**Table 2:** The emergent themes identified among 72 interviews on human-wildlife coexistence in northern Tanzania. The themes are specific to elephants (*Loxodonta africana*), but explore intersecting impacts of human-elephant and human-carnivore interactions. Each theme is presented with a detailed description and a representative quotation.

Emergent theme	Description	Example quotation
Threat to food security	Elephants pose a direct threat to the production, harvesting, or availability of food in farms.	“Now our main resource is livestock because elephants have destroyed the farms, we can’t rely on the farms. If we are lucky we have rains, we can grow enough crops, but elephants destroy the farms”
Threat to human safety	Elephants pose a direct threat to the safety of humans.	“We have several people who have died during elephant attacks. We also have a couple people who have been injured by elephants as well.”
Threat to societal well-being	Elephants contribute to the undercurrent of challenges that the community members face. They increase the difficulty of life in this landscape, sometimes to the point of threatening lives and lifestyles.	“There has been an increase in animals. Yesterday I was talking to someone about how in the next three years I don’t think that we will be able to do agriculture anymore because of them.”
Need for wildlife education	Community members need opportunities to learn how to help themselves. They want access to the knowledge that has been gathered regarding negative human-wildlife interactions, and best practices to mitigate the resultant impacts.	“We told you we want meetings, workshops, and seminars in our village [about wildlife], is that going to happen? Are we going to get that education and collaboration?”
Need for trust in government	Community members cannot rely on the support of the government (or its associated agencies) to prevent negative interactions with elephants or to recover afterward. They feel the government values animals above them.	“Sometimes people get hurt, these wild animals destroy our farms and livestock, and still no one is paying any attention. We don’t get any support from the government.”
Need for resources and solutions	Community members need assistance obtaining resources to protect themselves and their crops from elephants. They know what tools would be helpful, but they do not have a way to access them on their own.	“Our youth don’t sleep at night- they just stay awake to chase elephants away. Sometimes we try traditional methods like burning manure.”

**Table 3:** The frequency of occurrence for each of the six emergent themes identified among 72 interviews on human-wildlife coexistence in Northern Tanzania. Themes are also presented as a percentage of all theme occurrences.

Emergent theme	#	%
Threat to food security	94	23.0
Threat to human safety	19	4.6
Threat to societal well-being	49	12.0
Need for wildlife education	49	12.0
Need for trust in government	82	20.0
Need for resources and solutions	116	28.4

**Figure 4:**
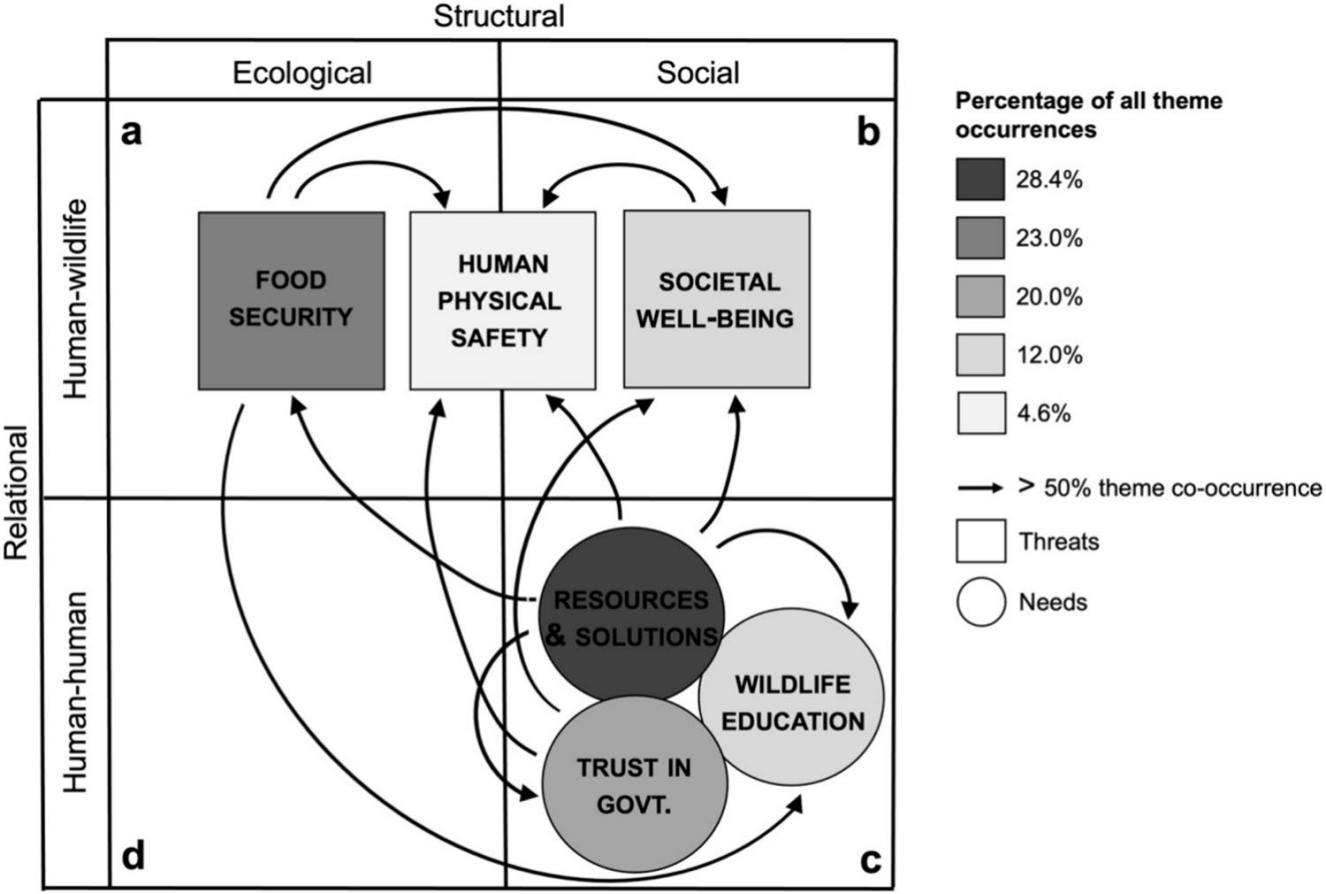
The six emergent themes, representing intersecting components within the two dimensions of complex systems in which human-wildlife coexistence is situated. Each theme is colored according to its frequency of occurrence, and the patterns of co-occurrence among the themes are represented by arrows. The theme at the origination of each arrow exhibits > 50% co-occurrence with the theme at the terminus of the arrow.

The theme ‘threats to food security’ contained components representing the intersection of ecological structures and human-wildlife interactions (Fig. 4a), while ‘threats to societal well-being’ represented the intersection of social structures and human-wildlife interactions (Fig. 4b). ‘Threats to human safety’ represented the intersection of components from both human-wildlife interactions and ecological structures as well as human-wildlife interactions and social structures (Fig. 4a, b). The themes ‘need for resources and solutions’, ‘need for wildlife education’, and ‘need for trust in government’ all contained components representative of the intersection of social structures and human-human interactions (Fig. 4c). No themes contained components representing the intersection between ecological structures and human-human interactions (Fig. 4d). All themes co-occurred with at least two others, but the need for resources and solutions was the only theme that co-occurred with all five additional themes (Fig. 4).

## Discussion

There is increasing evidence that a lack of understanding of the processes of feedback, intersection, and amplification among system components may be limiting the impact of human-wildlife coexistence research^19^. Therefore, explicit examination of such complex systems processes may promote the development of more effective coexistence solutions from research efforts^3,10^. Complex systems approaches may not be the best fit for all human-wildlife coexistence studies, as they add in layers of interactivity that may overcomplicate some highly-focused research objectives. However, during the process of conducting our interviews, the importance of a broadened complex systems approach to human-wildlife coexistence in our study became readily apparent. We did not introduce the topic of elephants, crop raiding, or any specific aspect of human-wildlife coexistence beyond that associated with carnivores. Despite that reality, almost three quarters of our participants voluntarily described impediments to coexistence with elephants, most of the time in the same phrase as they discussed those related to carnivores. Via this process of discovery, we learned that impediments to human-carnivore and human-elephant coexistence are fundamentally linked in this system.

Upon seeing the error in our research approach, we benefited from the logistical necessities of our interview process. As we had consolidated the two distinct sets of interview questions into one session, we had the data at hand to expand our analytical process beyond a single-component approach. We also recognize that this interview setup likely opened our participants up to broader considerations that may not have emerged if we had asked the carnivore-specific questions in isolation. Regardless, we initiated this research intending to interrogate human-carnivore interactions in isolation. We acknowledge that research approaches focused on a single component of a system, or even a single species, can provide great value in many circumstances^20^. However, our intention with this research was to explore impediments to human-carnivore coexistence in a complex system. Therefore, the single-guild approach we started with was not actually in alignment with the information we sought. We seek to promote greater transparency in human-wildlife coexistence research by openly discussing the process of adapting our methodology to a complex system approach and examining the systems-level understanding of impediments to coexistence such an approach can support.

After changing course to conduct a complex systems approach, we were able to explore the ways in which the six emergent themes (threats to food security, threats to human safety, threats to societal well-being, need for wildlife education, need for trust in government, and need for resources and solutions) contained components from more than one dimension of the complex system (Fig. 4) and the implications of those themes for impediments to human-wildlife coexistence.

We found that the coupled impacts of crop raiding and livestock depredation can erode both daily resources and emergency reserves for agropastoral food supplies, as highlighted in the theme ‘threats to food security’. Participants consistently identified both livestock and crops as important resources in their communities, but more commonly noted the threats elephants posed to the availability of those resources. Importantly, however, threats from elephants are not distinct from those associated with carnivores. Carnivore depredation of livestock also presents a profound direct threat to food security^49^. Depredation of livestock can cause substantial financial stress that limits abilities of livestock owners to purchase the supplementary food resources necessary for survival after elephant crop raiding^49^. Our participants noted this intersection, describing the substantial decrease in food security they experience when elephants destroy their crops and carnivores eat their livestock. One man from Mbuyuni summarized the double threat, stating “wild animals destroy crops, and carnivores such as lions, hyenas, and leopards eat our livestock. Especially in our area, elephants are destroying our crops which is why we depend on the livestock.” Thus, the ecological components of carnivore and elephant foraging behaviors have intersecting effects with human-wildlife interactions (Fig. 4a) that pose great risks to human food security.

The threats to human safety posed by elephants continue to build upon these impacts. While our interviews did not ask any participants to discuss the threats that elephants posed to their physical safety, many shared community fears and personal traumas deriving from their interactions with elephants. This pattern clearly indicates that they viewed the physical threats posed by elephants to be implicitly intertwined with livestock depredation by carnivores (Fig. 4a). Threats to human safety can also have psychological and social effects which can result in reduced capacity for individuals to respond to negative human-wildlife interactions, indicating a direct intersection of human-wildlife interactions and social structures (Fig. 4b)^40,50^. We found that this impact was also compounded by the intersection of carnivore and elephant interactions. For instance, when a male member of a family is injured or killed by an elephant, as was commonly described by our participants, the rest of the family must undergo rapid shifts in responsibility and workloads^50^. These individuals must maintain their other household duties and are therefore often working under conditions of stress and sleep deprivation^40,51,52^. The death or injury of a family member also brings additional emotional trauma, including grief, loneliness, and fear^51,52^. These intersections between human-wildlife coexistence and both social and ecological components of the system (Fig. 4a,b) are notable impediments to human-wildlife coexistence as a whole. The outcomes of such complex patterns of interplay can erode overall tolerance for wildlife and individual capacity to respond to the impacts of all species^53,54^.

These impediments were further elucidated in our assessment of the theme ‘threats to societal well-being’. Well-being is subjective and contextual and can encompass factors ranging from access to clean water to cultural identity and happiness^51,55^. One of the most notable threats to well-being referenced by our participants was fear that increasing rates of crop destruction by elephants might threaten the persistence of their way of life. For example, one participant told us “There has been an increase in elephants. Yesterday, I was talking to someone about how in the next three years I don’t think that we will be able to do agriculture anymore because of them.” While elephant populations may be at risk in other regions of sub-Saharan Africa, the elephant population in our study area has remained stable over the past few decades^33,56^. However, human populations have grown exponentially in the same time period, resulting in an increase in overlap between human agricultural lands and elephant ranges^31,56^. Additionally, changing climates are shortening growing seasons and driving stochasticity in water availability^31^. Together, these factors have resulted in increased rates of crop raiding by elephants during shorter growing seasons, thus limiting agricultural outputs and disrupting traditional livelihood practices^51,52^. The psychological effects of these stressors are a key social component of the system. Their intersection with human-wildlife interactions (Fig. 4b) can substantially reduce both individual capacity for participation in human-wildlife coexistence interventions, and willingness to do so, regardless of the species involved^51,53–55^.

We found that the needs for wildlife education, trust in government, and resources and solutions were further impediments to human-wildlife coexistence emerging from the intersection of social structure and human-human interaction components (Fig. 4c). Many participants expressed frustration that they had been communicating these needs consistently without seeing any changes. One man from Naitolia explained “we are tired because when you talk about something without any action, it is meaningless. It is supposed to be when you talk about something that an action should be taken.” Our participants expressed a desire to share in the wildlife knowledge they helped produce, to be valued by their government, and to have access to the resources they know exist. As these needs remained unmet, their patience for participating in continued human-wildlife coexistence research and interventions was quickly declining.

Our participants also identified an uneven distribution of education opportunities, in cases where the research findings were shared. They described how the government, wildlife authorities, and leaders in their communities had access to wildlife education and knowledge, but the rest of the communities were excluded from those learning opportunities. “The government has all the knowledge, and they’re supposed to bring this knowledge to the community. It’s very rare to see the government officials bringing it to us though,” explained another man from Esilalei. This hierarchical barrier in knowledge distribution is a source of concern in the pursuit of sustainable solutions for human-wildlife coexistence^49^. Leaving individuals in a cycle of threats to their well-being along with unmet needs can reinforce the antagonism they feel towards individuals who are perceived to be withholding that knowledge^51,57^.

A persistent need for wildlife education rendered our participants reliant upon government support. However, they expressed that they could not trust that their government would provide help either. Some individuals stated outright that this pattern was a result of the government valuing animals more than people, telling us “animals are more important than me,” and “it seems like the government cares more about animals than people.” The implications of this lack of valuation by their government are apparent in explanations like: “We’ve been participating and giving our feedback and recommendations, but no actions have been taken. So for us it doesn’t make sense to keep participating.” Exclusion from the spheres of knowledge and distrust in the reliability of wildlife authorities both contribute to decreases in tolerance for wildlife and willingness to participate in future coexistence interventions^42,49,54,58,59^. In addition, the types of knowledge and value inequities identified by our participants threaten the long-term sustainability of any interventions that are put in place^10,57^.

The need for resources and solutions continues to build upon the lack of willingness to participate in interventions. Even in instances in which the government offered support and showed intent to follow through on that promise, these efforts were limited by a lack of resources. “There are way more attacks than rangers, so there aren’t enough resources to help support the communities” one participant explained. At an individual level, the implications of limited resources for human-wildlife coexistence were even more apparent. When asked what strategies they use to deal with the challenges posed by wildlife, over half of our participants said that they did not have any at all. Many of these individuals mentioned trying “traditional methods” to deter animals from both crop raiding and livestock depredation. These methods typically consisted of staying awake at night so they could burn manure, use the bottom of buckets as drums, and yell at the animals. They emphasized an understanding of the ineffectiveness of that approach, particularly over long time periods as “the elephants get used to it so they know that it’s nothing and come and destroy your crops.” Importantly, night is the time period in which both the risk of livestock depredation and crop raiding are highest^24,60^. Therefore, individuals must simultaneously attempt to protect their crops from elephants and be prepared to chase off a potentially depredating carnivore. The consistent lack of sleep resulting from this combined threat has been linked to increased rates of alcoholism^51^, exposure to malaria^61^, mental health morbidity^52,62^, and reduced school performance for students^63^. Individuals experience such impacts in addition to the direct physical dangers associated with defending livestock and crops, which is particularly risky at night^48,64^. Therefore, the persistent need for resources and solutions may increasingly reduce community members’ physical, emotional, and psychological capacity to participate in coexistence interventions. This limited capacity on top of similarly eroded willingness to participate may severely impede human-wildlife coexistence.

No themes contained components from both ecological structures and human-human interactions (Fig. 4d). This result indicates that the human-human interactions, which constitute a substantial impediment to human-wildlife coexistence^4,7^, are unlikely to be elucidated via research that centers on ecological structures. Ecological research tends to focus on the development of technical solutions to the impacts of wildlife^19,41^. While such efforts have value in and of themselves, they cannot provide insights on the interactions that arise from misalignment between the goals, values, and needs of groups of people^41,43^. Thus, the lack of research that focuses on these social structures is likely a contributing factor to the intractability of human-wildlife coexistence^41,65^. The addition of knowledge drawn from the social sciences will be necessary to develop effective solutions to the human-human impediments to human-wildlife coexistence^4,42,66^. Moreover, this knowledge must be applied in explicit consideration of the social components inherent to the complex system^42,57,65^.

We identified two key patterns from the intersections of themes across the system as a whole. The first is that the need for resources and solutions appears to be foundational in this system. This need was not only the most common theme, but also was the only one that exhibited > 50% co-occurrence with all five other themes (Fig. 4). Therefore, addressing this particular need may have cascading effects across the system. The importance of reliable resources and solutions has been consistently documented in research on human-wildlife coexistence^22,41,53^. In fact, technical interventions designed to provide such solutions are among the most commonly recommended approaches to promote coexistence^67^. However, the effects of structured resource provision on the social impediments to coexistence remain in need of further exploration^10,43,67^. The second notable pattern that emerged from the broad perspective is that no single theme existed in isolation. Each theme was connected via co-occurrence with at least two others. This interconnectedness emphasizes the complexity of this system. Further, it reveals the importance of research that can provide better understanding of the ways in which components interact within such complex systems.

## Conclusions

Complex systems approaches are challenging to design and carry out^3,6^. CHANS studies, in particular, often require interdisciplinary research teams, study designs that bridge traditionally-siloed social and ecological knowledge-bases, and complicated logistics for data collection^2,4,7^. Furthermore, such research is often difficult to publish in an academic system that rewards focused research on single species and components^68–70^. We also recognize that focused research programs intended to reveal depth and nuance within a single component of a system have value. However, the goal of such research programs is most often purported to be the provision of knowledge that can inform coexistence interventions^68^. These interventions must consider interactions among the diverse components of the complex system to be effective^3,10^. Therefore, basing interventions on human-wildlife coexistence research that only considers one component of the system is unlikely to produce solutions that are effective over the long-term^10,43,49,66^. In our case, the single-component approach provided insights into which carnivore species require the most targeted interventions for livestock depredation. However, the oversimplified design failed to reveal that human-carnivore coexistence is unlikely to be achieved without concurrent interventions around human-elephant coexistence and repairing relationships of trust and support between communities and the government.

Currently, the conservation field lacks insight into the ways in which components of complex systems may interact^18,19^. As human-wildlife coexistence is situated within complex systems, this knowledge gap serves as a notable impediment to the development of effective interventions and is perpetuated by patterns of system simplification and a lack of transparency around failures in research design.^3,6,57^. We seek to bring attention to this pattern by directly addressing the limitations of our initial approach and including it alongside our improved methodology centered on complex systems. We encourage others to embrace transparency and open-mindedness in the research process through careful consideration of whether chosen approaches align with the outputs sought and the complexity of the system. Further, we advocate for similar considerations by funding bodies, governments, NGOs, and all other institutions that share and support the goal of human-wildlife coexistence. Most importantly, we urge researchers in the human-wildlife coexistence field to recognize when their approach is out of alignment and change course. This self-awareness and willingness to accept failure enables us to not be blinded by our initial assumptions and devise a more complete understanding of the systems under study.

## Supplementary Information

Supplementary Information.

## Data Availability

The datasets generated and analyzed during the current study are not publicly available to protect the privacy of participants, due the personal nature of the topics discussed. However, data can be made available from the corresponding author on reasonable request.
