# Supplementary material for: Transparency and adaptability aid in realigning the complexity of objectives, approaches, and systems in human-wildlife coexistence research
Source: Sci Rep. 2024 Sep 17;14:21670. doi: 10.1038/s41598-024-69563-5 (PMC11408497; doi:10.1038/s41598-024-69563-5)
Supplement: Supplementary file 1 — Supplementary Information. [file 41598_2024_69563_MOESM1_ESM.docx]

**APPENDIX I**

*Complete interview question set*

Date:

Ward:

Village:

Sub-Village:

Boma #:

Sex of Resp:

GPS:

Background

1. Can you describe what is your daily life is like?
2. What do you like about this community? (What is important to you in your community)

Resources

1. What resources are available in this area?
   1. Do community members use these resources?
2. How do these resources help community members to improve their well-being?
3. Are these resources distributed equally among community members?
   1. If YES, how?
   2. If NO, why not?

Livestock predation

1. How often is each carnivore species at your boma attacking (or trying to kill) livestock, and not attacking livestock?

*Lions (simba)*

|  | Very often  (Mara nyingi sana) | Often  (Mara nyingi) | Sometimes  (Mara chache) | Rarely  (Mara chache sana) | Never  (kamwe) | How many times/week, month? | Reason for not trying to kill livestock? |
| --- | --- | --- | --- | --- | --- | --- | --- |
| Mara ngapi anakuja na kula mifugo  (At the boma -try to kill livestock) |  |  |  |  |  |  | N/A |
| Mara ngapi anakuja na hali mifugo  (At the boma – not try to kill livestock) |  |  |  |  |  |  |  |

*Hyenas (fisi)*

|  | Very often  (Mara nyingi sana) | Often  (Mara nyingi) | Sometimes  (Mara chache) | Rarely  (Mara chache sana) | Never  (kamwe) | How many times/week, month? | Reason for not trying to kill livestock? |
| --- | --- | --- | --- | --- | --- | --- | --- |
| Mara ngapi anakuja na kula mifugo  (At the boma -try to kill livestock) |  |  |  |  |  |  | N/A |
| Mara ngapi anakuja na hali mifugo  (At the boma – not try to kill livestock) |  |  |  |  |  |  |  |

*Leopards (chui)*

|  | Very often  (Mara nyingi sana) | Often  (Mara nyingi) | Sometimes  (Mara chache) | Rarely  (Mara chache sana) | Never  (kamwe) | How many times/week, month? | Reason for not trying to kill livestock? |
| --- | --- | --- | --- | --- | --- | --- | --- |
| Mara ngapi anakuja na kula mifugo  (At the boma -try to kill livestock) |  |  |  |  |  |  | N/A |
| Mara ngapi anakuja na hali mifugo  (At the boma – not try to kill livestock) |  |  |  |  |  |  |  |

*Jackal (mbweha)*

|  | Very often  (Mara nyingi sana) | Often  (Mara nyingi) | Sometimes  (Mara chache) | Rarely  (Mara chache sana) | Never  (kamwe) | How many times/week, month? | Reason for not trying to kill livestock? |
| --- | --- | --- | --- | --- | --- | --- | --- |
| Mara ngapi anakuja na kula mifugo  (At the boma -try to kill livestock) |  |  |  |  |  |  | N/A |
| Mara ngapi anakuja na hali mifugo  (At the boma – not try to kill livestock) |  |  |  |  |  |  |  |

*Other*

|  | Very often  (Mara nyingi sana) | Often  (Mara nyingi) | Sometimes  (Mara chache) | Rarely  (Mara chache sana) | Never  (kamwe) | How many times/week, month? | Reason for not trying to kill livestock? |
| --- | --- | --- | --- | --- | --- | --- | --- |
| Mara ngapi anakuja na kula mifugo  (At the boma -try to kill livestock) |  |  |  |  |  |  | N/A |
| Mara ngapi anakuja na hali mifugo  (At the boma – not try to kill livestock) |  |  |  |  |  |  |  |

1. In the past year (2017-2018), how many livestock have you lost to carnivore depredation?

|  | (Estimated) number | Responsible carnivore |
| --- | --- | --- |
| Cattle |  |  |
| Shoats |  |  |
| Donkeys |  |  |
| Chickens |  |  |
| Other |  |  |

1. Which carnivore species do you think poses the greatest risk to your livestock or livelihood?

Wildlife Conservation

1. Do you understand the term wildlife conservation?
   1. What are the biggest challenges for wildlife conservation this area?
   2. How has wildlife conservation changed in the past 10 years?
2. Do community members participate in decisions regarding wildlife?
   1. If so, who participates and how?
3. What do you think are the benefits that wildlife conservation has for the community?
   1. Have you benefited financially from wildlife conservation?
4. What are the difficulties wildlife conservation causes for the community?
   1. How do you overcome those difficulties?
   2. Who in your community faces the most difficulties?

Additional Comments

1. Is there anything else you think we should discuss that was not covered? Do you have any other thoughts or recommendations that you would like to share?
